# Psychometric and qualitative assessment of the Client Satisfaction Questionnaire-8 (CSQ-8) among Syrian mental health and psychosocial support service users

**DOI:** 10.1192/bjo.2026.11013

**Published:** 2026-04-28

**Authors:** Michael McGrath, Ahmed El-Vecih, Gülşah Kurt, Salah Addin Lekkeh, Wael Yasaki, Ammar Beetar, Simon Rosenbaum, Ruth Wells

**Affiliations:** Discipline of Psychiatry and Mental Health, University of New South Wales, Sydney, Australia; Hope Revival Organization, Gaziantep, Türkiye; School of Psychology, University of New South Wales, Sydney, Australia

**Keywords:** Mental health services, patients and service users, refugee mental health, conflict and displacement, MHPSS

## Abstract

**Background:**

Patient satisfaction is an important indicator of mental healthcare quality. The Client Satisfaction Questionnaire-8 (CSQ-8) is widely used, but its psychometric performance and contextual relevance have not been examined in humanitarian settings.

**Aims:**

To assess the reliability and validity of the CSQ-8 in a sample of displaced and conflict-affected Syrian mental health and psychosocial support (MHPSS) service users.

**Method:**

Structured telephone interviews were conducted with Syrian MHPSS service users in North-West Syria. Psychometric analysis assessed the internal consistency and construct validity of the CSQ-8. Convergent validity with therapeutic alliance was examined using the Working Alliance Inventory Short Revised. Item comprehension and contextual relevance were examined using open-ended qualitative responses.

**Results:**

Among 337 participants, the CSQ-8 demonstrated strong internal consistency (*ω* = 0.92), but suboptimal model fit (comparative fit index 0.922; Tucker–Lewis index 0.890; root mean square error of approximation 0.132). Convergent validity with therapeutic alliance confirmed the importance of service user–practitioner relationships to satisfaction. Item 4 (‘Would you recommend the service?’) and item 8 (‘Would you return to the service?’) showed large ceiling effects and weaker psychometric properties. Qualitative data indicated responses to these items were shaped by community stigma, low expectations and a lack of alternative services, rather than satisfaction. A secondary analysis of the CSQ-4 showed good reliability (*ω* = 0.81) and improved model fit (comparative fit index 0.993; Tucker–Lewis index 0.980; root mean square error of approximation 0.065).

**Conclusions:**

In this humanitarian setting, items about a willingness to recommend and intent to return to a service may lack conceptual validity and should be used cautiously. Shorter forms such as the CSQ-4 may be a useful alternative.

Patient satisfaction is a widely recognised indicator of high-quality, person-centred care.^1,2^ As a patient-reported measure, satisfaction complements clinical outcome indicators by providing insights into individuals’ experiences and their perceptions of care quality. Ongoing efforts to strengthen quality of care in low- and middle-income countries have prioritised the measurement of health system inputs, such as the number of facilities and service coverage, with comparatively less attention given to processes of care and patient-reported indicators, like satisfaction.^2,3^ Nonetheless, measuring satisfaction is important for assessing programme acceptability, guiding quality improvement and reorientating care toward patient and community priorities.^4^ Higher satisfaction is associated with greater confidence and trust in health systems, and can promote engagement and retention in services.^5,6^

## Measuring satisfaction

Despite its importance, patient satisfaction remains a theoretically contested concept, with ongoing debate about how it should be defined and measured.^1,5^ Early conceptualisations drew heavily on consumer and expectancy theories, framing satisfaction as the fulfilment of needs and expectations or in terms of the value and utility derived from healthcare.^7,8^ However, expectations are not fixed judgements, but are dynamic, context-specific and shaped by previous experiences of healthcare.^4^ Evidence from multiple healthcare settings and populations indicates that satisfaction is most consistently associated with patient perceptions of care quality, with relational and interpersonal aspects of care, including therapeutic alliance, most prominent, alongside other factors such as technical quality, physical environment and accessibility.^9^

Methodological challenges to measuring satisfaction compound these conceptual issues. Social desirability bias and acquiescence bias can inflate patient-reported ratings.^10^ In low-resource or highly unequal health systems, a dependence on a limited number of available services may leave patients reluctant to provide negative feedback because of concerns about compromising access to care. Expectations of health services are often low in humanitarian settings, because of previous experiences of poor quality of care, limited agency, discrimination and power asymmetries in care relationships.^2^ As a result, patient-reported satisfaction is often high, despite objectively poor quality of care.^2,11^ Satisfaction scores are frequently negatively skewed, suffering from ceiling effects that overstate patient experiences, mask variation across subgroups and limit the ability of satisfaction measures to detect meaningful change.^9^

## Client Satisfaction Questionnaire-8

The Client Satisfaction Questionnaire-8 (CSQ-8) is the most widely used instrument for measuring patient satisfaction in mental health services.^12^ Originally developed in a sample of mental health out-patients in the USA, it has since been implemented in in-patient, out-patient and community mental health services globally.^12,13^ Validation studies with mental health patients have primarily been conducted in high-income countries.^14–16^ These studies have demonstrated the instrument’s validity and reliability, confirmed a unidimensional structure, and reported pronounced ceiling effects and negatively skewed satisfaction scores.

Despite this evidence being confined to high-income settings, the CSQ-8 is increasingly used in global mental health research. It has been implemented as a complete instrument or in a shortened form, primarily within process evaluations of intervention feasibility and acceptability, including studies of refugee mental health services, child and adolescent care, substance use treatment and gender-based violence programming in low- and middle-income countries.^17–23^ The instrument’s validity and reliability in low-resource settings or among populations affected by humanitarian crises have not been examined. Without adaptation or assessment of cross-cultural equivalence or cultural relevance, standardised instruments developed in high-income countries, like the CSQ-8, may fail to capture culturally specific understandings of psychological distress, patient–provider relationships and care-seeking behaviours.^11^ Misspecified or misinterpreted measures risk producing inaccurate assessments of patient experiences and diverting limited resources toward inappropriate or poorly implemented services.^4,24^ As a result, satisfaction measures require culturally grounded adaptation, using both qualitative and psychometric methods to ensure that tools are valid and reliable across settings.^11^ In this study, we aim to (a) test the psychometric properties of the CSQ-8 in a sample of displaced Syrians attending mental health and psychosocial support (MHPSS) services in North-West Syria; and (b) qualitatively examine the instrument’s contextual and cross-cultural validity in this setting.

## Method

### Setting

The Syrian Civil War has left 16.7 million people in need of humanitarian assistance, representing one of the world’s largest humanitarian crises.^25^ At the time of our data collection, North-West Syria was under opposition control and experiencing ongoing conflict. The formal health system had collapsed, and mental healthcare was provided through an informal network of Syrian MHPSS organisations operating under ongoing insecurity and with limited international assistance. MHPSS adopts a coordinated, multisectoral approach to protecting and promoting the psychosocial well-being and mental health of people living in humanitarian settings.^26^ This includes specialist psychiatric and psychological care delivered by mental health professionals, alongside focused psychosocial interventions and community-based support delivered by non-specialists. During our data collection, the region was affected by the 2023 Türkiye–Syria earthquakes, exacerbating the humanitarian crisis and resulting in widespread injury, loss of life and the destruction of housing and infrastructure.^27^

### Participants

The Caring for Carers project is a mixed-methods, quasi-experimental study of a supportive supervision intervention for MHPSS workers in North-West Syria and Türkiye.^28^ The primary aim of the project was to measure the impact of supportive supervision for MHPSS workers on practitioner well-being. Between April 2022 and February 2024, additional data were collected from the service users of these MHPSS practitioners to understand their experiences of care.

This paper presents a secondary analysis of service user data collected as part of the project. We include data from service users who attended an MHPSS session with one of the 38 non-specialist Syrian practitioners in North-West Syria who participated in the Caring for Carers project. Eligible participants met the following criteria: (a) Syrian nationals, (b) aged 18 years and older, (c) living in North-West Syria, (d) who had attended an MHPSS session in the past 3 weeks (either as a service user themselves or as an accompanying parent or carer) and (e) provided informed consent. Participants were a mix of local and internally displaced Syrians living in urban areas, refugee camps and informal settlements.

All participants attended an MHPSS session provided by local Syrian practitioners working with one of the nine participating MHPSS organisations. These sessions involved individual and group-based psychosocial support and counselling, including task-shifted, guided interventions delivered by trained and supervised non-specialist practitioners. All care was delivered independently by participating organisations as part of routine service provision.

Individual participants cannot be identified in the manuscript, and all data have been anonymised. The authors assert that all procedures contributing to this work comply with the ethical standards of the relevant national and institutional committees on human experimentation and with the Helsinki Declaration of 1975, as revised in 2013. All procedures involving human participants were approved by ethics committees at the University of New South Wales, Australia (approval number HC210824) and Koç University, Türkiye (approval number 2021.395.IRB3.182).

### Data collection and measures

Because of the highly unstable setting, recruitment followed a pragmatic, non-probabilistic strategy through 38 MHPSS practitioners enrolled in the study in North-West Syria. Random sampling of service users was not feasible, and structured telephone interviews were the only safe and practical method of data collection given the safety and security concerns in the region. To reduce systematic time and day-of-week selection effects, morning or afternoon time blocks and weekdays were randomly allocated to practitioners each month. Participating organisations provided the telephone contact details of eligible service users who had attended sessions with these practitioners during this time block. Interviews were conducted up to 3 weeks after the session. The 3-week eligibility window was chosen to minimise recall bias while allowing sufficient time to contact participants and schedule a telephone interview. To protect confidentiality and safety, individuals were not contacted if doing so might risk disclosing their attendance at MHPSS services to others.

During recruitment, research assistants explained that participation was voluntary and that neither their MHPSS practitioner nor the service would be informed of their participation. Verbal informed consent was obtained from all participants, as written consent was not feasible. Interviews were conducted in Arabic by trained Syrian research assistants living in the region. Data were input into KoboToolbox, a secure digital platform.^29^ Data collection was suspended for 4 months following the earthquake.

As a secondary analysis of data collected within a wider project, no formal sample size calculation was conducted. However, the final sample exceeds recommended minimum sample sizes for confirmatory factor analysis and structural equation modelling of short instruments.^30^

#### CSQ-8

All participants were administered the CSQ-8,^13^ an eight-item measure of satisfaction that uses a four-point Likert scale. Items are summed to produce a total satisfaction score ranging from 8 to 32, with higher scores indicating greater satisfaction. Minor adaptations were made to the Arabic version of the CSQ-8, including changing formal written Arabic to conversational Syrian Arabic to enhance comprehension during telephone interviews. Item wording was also adjusted to ensure responses related to the practitioner delivering care rather than to other services received within integrated MHPSS services (see survey instrument in the Supplementary Material). Bilingual researchers independently conducted forward and backward translation, with discrepancies reconciled through discussion within the team. Open-ended, follow-up questions were added to individual items to examine item comprehension and qualitatively explore service user satisfaction.

#### Working Alliance Inventory Short Revised

A subset of participants also completed the Working Alliance Inventory Short Revised (WAI-SR), a 12-item measure of therapeutic alliance between client and practitioner.^31^ The WAI-SR was initially included in the full survey instrument. However, because of the challenges of data collection in this setting, the measure was discontinued after 24 weeks, to minimise participant burden and reduce interview duration. The WAI-SR comprises three subscales assessing agreement on treatment goals, agreement on task collaboration and the emotional bond between client and practitioner. Items are rated on a five-point Likert scale and summed to generate total and subscale scores. Higher scores reflect stronger perceived working alliance.

### Statistical analysis

Quantitative analysis was conducted in R, version 4.4.2 for macOS (R Foundation for Statistical Computing, Vienna, Austria; http://www.r-project.org/) and RStudio, version 2025.05.0+496 (Posit Software, Boston, Massachusetts, USA; https:posit.co/download/rstudio-desktop/). Descriptive statistics (mean, median and s.d.) were calculated for satisfaction scores, sociodemographic variables and service information. As service users were clustered within practitioners and intraclass correlation coefficients (ICCs) for satisfaction scores indicated moderate clustering (ICC = 0.13), all analyses adjusted for clustering at the practitioner level. Mean differences in satisfaction scores between binary variables (gender and proxy participants versus service user participants) were estimated using linear regression, with 95% confidence intervals calculated using cluster-robust standard errors. Mean satisfaction scores by the number of sessions with this practitioner were analysed as an ordinal variable, using linear regression and cluster-robust standard errors.

Internal consistency was assessed using McDonald’s omega. Item-level performance was examined using corrected item-total correlations and Cronbach’s α if item deleted. Sampling adequacy and suitability for factor analysis were evaluated using the Kaiser–Meyer–Olkin (KMO) statistic (KMO ≥0.60 acceptable; ≥0.80 good) and Bartlett’s test of sphericity. Exploratory factor analysis was conducted to explore dimensionality, with factor retention guided by scree plots and eigenvalues >1, and factor loadings ≥0.40 considered acceptable. Confirmatory factor analysis tested a one-factor model with all eight items, with standard errors adjusted for clustering of service users within practitioners. Model fit was evaluated using the non-significant χ^2^ test, alongside standard indices and their recommended cut-offs: comparative fit index (CFI) (CFI ≥0.95 good; ≥0.90 acceptable), Tucker–Lewis index (TLI) (TLI ≥0.97 good; ≥0.95 acceptable), standardised root mean square residual (SRMR) (SRMR ≤0.05 good; ≤ 0.10 acceptable) and root mean square error of approximation (RMSEA) (RMSEA ≤0.05 good; ≤0.08 acceptable).^32^

Convergent validity was evaluated by examining the relationship between satisfaction scores and therapeutic alliance (WAI-SR), based on a hypothesised positive relationship between these constructs. Associations were assessed using Spearman rank correlations and structural equation modelling, with standard errors adjusted for clustering. In structural equation models, therapeutic alliance was specified as a latent factor indicated by the WAI-SR task, goals and bond subscales.

Open-ended qualitative responses were analysed using content analysis to assess the face and content validity of individual items. Qualitative responses were coded for item comprehension, cross-cultural equivalence and contextual relevance. All qualitative analysis was conducted using NVivo, release 14.20.0(13) (Lumivero, Burlington, Vermont, USA; https://lumivero.com/products/nvivo/).

### Secondary analysis

The CSQ-8 was prespecified in the wider study protocol and administered to all participants. However, following psychometric and qualitative analyses, a secondary analysis of the CSQ-4 was conducted in the same sample of service users. The CSQ-4 is a shortened version of the CSQ-8, comprising only items 3, 6, 7 and 8. Total scores range from 4 to 16, and the instrument has been validated in other settings.^33^

## Results

In total, 337 MHPSS service users completed interviews (56% female, mean age: 31 years). Participants had attended a median of four sessions with this MHPSS practitioner (range 1–30), and 28 people (8.1%) were attending their first MHPSS session with this practitioner. Overall, satisfaction was high, with a mean CSQ-8 score of 27.5 (range = 11–32) (Table 1). CSQ-8 scores were significantly lower in women than men (mean difference = 1.1 [95% CI: 0.2–2.1]) (Table 2). No difference in satisfaction scores was observed between the 29 participants (8.6%) who completed proxy interviews as the parent or caregiver of a service user they had accompanied to the session and service users themselves. CSQ-8 scores increased with the number of sessions attended with the same practitioner (β = 0.62, *p* = 0.003).

**Table 1:** Descriptive statistics, reliability and model fit statistics for the Client Satisfaction Questionnaire-8 (CSQ-8) and the Client Satisfaction Questionnaire-4 (CSQ-4)

Statistic	CSQ-8	CSQ-4
Mean score (s.d.)	27.5 (3.3)	13.7 (1.6)
Range	11–32	5–16
Cronbach’s α	0.88	0.77
McDonald’s omega	0.92	0.81
Comparative Fit Index	0.922	0.993
Tucker–Lewis Index	0.890	0.980
Root mean square error of approximation (RMSEA)	0.132	0.065
RMSEA [90% CI]	[0.110–0.154]	[0.000–0.147]
Standardised root mean square residual	0.057	0.017
χ^2^ test (*p*-value)	122.8 (<0.001)	4.6 (0.103)

**Table 2:** Variation in mean Client Satisfaction Questionnaire-8 (CSQ-8) and Client Satisfaction Questionnaire-4 (CSQ-4) scores by demographic and service-use characteristics

Variable	*n* (%)	CSQ-8 (s.d.)	CSQ-4 (s.d.)
Gender			
Male	149 (44.2)	28.1 (3.3)	13.9 (1.7)
Female	188 (55.8)	27.0 (3.3)	13.6 (1.5)
Mean difference [95% CI]	–	1.1 [0.2 to 2.1]	0.3 [−0.2 to 0.7]
Sessions with this practitioner			
1 session	28 (8.3)	27.1 (2.8)	13.2 (1.6)
2–3 sessions	129 (38.3)	26.8 (3.7)	13.3 (1.7)
4–5 sessions	104 (30.9)	27.8 (3.1)	14.0 (1.4)
6+ sessions	76 (22.6)	28.3 (3.0)	14.3 (1.3)
Proxy interviews			
Service user participant	29 (8.6)	27.4 (3.3)	13.7 (1.6)
Parent or carer participant	308 (91.4)	27.9 (3.6)	13.9 (1.8)
Mean difference [95% CI]	–	0.5 [−1.0 to 1.9]	0.2 [−0.5 to 0.9]

The CSQ-8 demonstrated strong internal consistency (ω = 0.92). Sampling adequacy was good (KMO = 0.87) and Bartlett’s test of sphericity statistically significant (*χ* ^2^ = 1507.8(28), *p* < 0.001). Exploratory factor analysis supported a single factor structure accounting for 49% of the variance and standardised factor loadings ranged from 0.38 to 0.95. In confirmatory factor analysis, the unidimensional model failed to meet the recommended thresholds of acceptable model fit across all indices (CFI = 0.922, TLI = 0.890, SRMR = 0.057; RMSEA = 0.132, 90% CI [0.110–0.154]).

Convergent validity was supported by moderate to strong positive correlations between the total CSQ-8 score and total WAI-SR score (ρ = 0.56), as well as the three WAI-SR subscales: goal (ρ = 0.41), task (ρ = 0.53) and bond (ρ = 0.29), in the 115 participants who had been administered both instruments. These associations were confirmed in structural equation modelling, which demonstrated a strong relationship between latent therapeutic alliance and CSQ-8 scores (standardised *β* = 0.536). The task (λ = 0.92) and goal (λ = 0.81) subscales showed stronger loadings on the latent therapeutic alliance factor than the bond subscale (λ = 0.38).

Psychometric analyses consistently identified item 4 (‘Would you recommend our program to a friend?’) and item 8 (‘If you were to seek help again, would you come back to our service?’) as underperforming. Both items displayed the largest ceiling effects, with 73.3 and 85.8% of participants respectively endorsing the most satisfied Likert response category (Table 3). Their corrected item-total correlations (0.50 and 0.41) and standardised factor loadings (0.51 and 0.38) were the lowest in the instrument. Alpha-if-item-deleted analysis also indicated marginal increases in internal consistency if these items were removed.

**Table 3:** Item-level statistics for the Client Satisfaction Questionnaire-8

Item	Mean (s.d.)	Ceiling effect^a^ (%)	Alpha if item deleted	Corrected item-total correlations	Standard factor loading
1: How would you rate the quality of the service you received?	3.4 (0.5)	40.1	0.87	0.59	0.592
2: Did you get the kind of service you wanted?	3.3 (0.6)	38.9	0.86	0.70	0.660
3: To what extent has the service met your needs?	3.0 (0.6)	15.7	0.87	0.59	0.561
4: If a friend needed similar help, would you recommend our service?	3.6 (0.9)	73.3	0.88	0.50	0.507
5: How satisfied are you with the amount of help you received?	3.5 (0.5)	46.6	0.85	0.80	0.949
6: Have the services you received helped you to deal more effectively with your problems?	3.4 (0.6)	45.1	0.85	0.75	0.783
7: In an overall, general sense, how satisfied are you with the service you received?	3.5 (0.5)	47.2	0.85	0.79	0.934
8: If you were to seek help again, would you come back to our service?	3.9 (0.4)	85.8	0.89	0.41	0.382

a. Percentage of respondents endorsing the highest Likert category.

Qualitative responses to these two items were examined to understand the reasons for their poor performance. Two recurring issues undermined item comprehension and contextual relevance: a reluctance to recommend the service was influenced by mental health stigma, and the intent to return reflected limited options or alternative services.

### Item 4: recommendation influenced by mental health stigma

Among the 41 participants who would not recommend the service, 34 (9.9% of the total sample) described mental health stigma as the reason rather than service dissatisfaction. This response pattern was gendered: 32 of these 34 participants who were concerned about stigma were women. Measurement invariance testing did not support equivalence of the measurement model by gender. In ordinal logistic regression adjusting for overall satisfaction (total CSQ-8 score excluding item 4), gender was a strong predictor of response to this item (χ^2^(1) = 31.6, *p* < 0.001) with men having higher odds of endorsing the item at equivalent satisfaction levels (*β* = 1.82; odds ratio = 6.2).

In open-ended qualitative responses, many women reported attending sessions discreetly or in secrecy and were concerned that recommending the service would disclose their own attendance.

‘Because here in the camps, it’s important to keep things to yourself. It’s better not to advise anyone to seek mental health support or tell anyone to attend psychological therapy, because people are harsh and will ruin a woman’s reputation [بطالعوا سمعة]. They’ll say a woman is crazy [مجنونة] and that she’s going to a madhouse [مشفى مجانين]. … I personally asked to attend secretly, and no one knows.’

Others explained that recommending a service to others was potentially insulting:

‘…many people have a negative perception of visiting a psychosocial support centre and attending sessions. They think that those who visit such centres are mentally ill [مريض نفسياً]. Therefore, I can’t recommend it to anyone because they might think I’m implying they’re crazy [مجنون] or have a nervous illness [مرض عصبي], which might upset them.’

### Item 8: intent to return reflected relational satisfaction under constrained choice

Among the 46 participants in the lowest decile of CSQ-8 scores, 45 selected a response to item 8 indicating that they would return to the service. Qualitative responses for these participants revealed that for many the decision to return reflected genuine satisfaction with relational aspects of care, even when their primary concerns remained unresolved. This was described in terms of positive features of the therapeutic relationship that facilitated trust, such as respectful treatment, attentive and supportive practitioners, and guaranteed confidentiality, alongside the provision of material support or feeling temporary emotional relief. One man described:

‘My employment is irregular. I am displaced and live in a village where I don’t know anyone, and I have problems at home and feel constantly tense and upset. [The practitioner] taught me problem-solving strategies and how to deal with stress, such as exercising and breathing deeply. …I leave the sessions feeling that things are okay, but after a while I return to how I was before. My situation remains the same. … [I would return because] it is enough that there is someone who helps you, listens to you, and understands how you feel. The counsellor is respectful and puts in all his effort to help.’

The intent to return was also shaped by low expectations of what their practitioner could provide and the lack of alternative services and treatment options in this setting. Participants frequently described poverty, displacement, ongoing conflict and the recent earthquake as the origin of their psychological distress. They understood these complex issues as beyond the capacity of their practitioner to resolve and expressed appreciation for the efforts of practitioners who ‘try to offer everything they have’ in the face of these challenges. One participant, whose father attended MHPSS sessions for conflict-related trauma, anxiety and somatic symptoms, explained:

‘My father’s condition improved, even if just a little … Yes, we would return because just having someone who listens, understands, and helps you to improve your condition is enough.’

For other participants, the intent to return despite otherwise unsatisfactory experiences reflected the absence of other mental health services and treatment options. One man described:

‘I suffer from obsessive–compulsive disorder. The counsellor taught me how to breathe deeply and just gave me advice. He gave me everything he could but that was all he was able to do. … I don’t have any other option.’

Some participants interpreted the item more broadly, describing the health centre as a whole, rather than the MHPSS service specifically. In these cases, many planned to return to obtain psychotropic medications that had been unavailable during their initial visit, for referral to specialist psychiatric or psychological care, or to access other health services and social support.

### Secondary analysis

Given the observed psychometric properties and qualitative evidence of misinterpretation, we conducted a secondary, exploratory analysis of the reduced CSQ-4. Satisfaction remained high, with a mean score of 13.7 (range 5–16) (Table 1). CSQ-4 scores increased with attendances at more sessions with this practitioner (*β* = 0.45, *p* < 0.001). However, unlike the CSQ-8, no gender differences were observed between women and men (mean difference: 0.3 [95% CI: −0.2 to 0.7]).

Internal consistency was good (*ω* = 0.81), model fit met established cutoffs for all indices (CFI = 0.993; TLI = 0.980; SRMR = 0.017; RMSEA = 0.065 [90% CI: 0.000–0.147]), and convergent validity was comparable to the CSQ-8: total WAI-SR score (*ρ* = 0.54), goal (*ρ* = 0.40), task (*ρ* = 0.51) and bond (*ρ* = 0.30). As with the CSQ-8, structural equation modelling demonstrated a strong association between latent therapeutic alliance and CSQ-4 scores (standardised *β* = 0.519), with task and goal subscales similarly showing stronger loadings on the latent alliance factor than the bond subscale (*λ* = 0.91, 0.83 and 0.39, respectively).

## Discussion

This study examined the validity, reliability and contextual relevance of the CSQ-8 in a sample of displaced and conflict-affected Syrian MHPSS service users. Participants reported high overall satisfaction with routine care, and the CSQ-8 showed strong internal consistency and convergent validity with therapeutic alliance. However, item-level analysis and qualitative findings revealed limitations in conceptual equivalence and interpretability in this setting.

Contextual and cultural factors partially explain the poor performance of items 4 and 8. Seventy-three per cent of participants indicated that they would definitely recommend the service (item 4). Among the small proportion (12%) who would not, qualitative responses revealed that this reluctance was driven by stigma rather than dissatisfaction or negative experiences of care. In Syria and other Arabic-speaking countries, stigma surrounding mental health often reflects fear of social judgement, reputational damage and family shame, particularly for women.^34,35^ Concerns about disclosing mental health service use may be intensified in camp settings due to a lack of privacy and overcrowded living conditions.^36^ In this context, community stigma and fear of disclosure introduced response bias in item 4, especially for women. Without this bias, ceiling effects would have been even larger, further reducing the instrument’s discriminative power.

Item 8 showed the largest ceiling effect, with nearly all participants reporting they would return to the service, even when otherwise dissatisfied. While ceiling effects are a known limitation of satisfaction measures, their impact might be greater in humanitarian settings where expectations are low and choices are limited.^2^ Qualitative findings suggested multiple explanations for these low expectations. For some, their distress was understood as a consequence of complex psychosocial and structural challenges that MHPSS services are not positioned to address, such as displacement, poverty, ongoing conflict and the recent earthquake. Other service users presented with severe or persistent mental health disorders that are beyond the training and capacity of non-specialist practitioners. Task-shifted MHPSS interventions delivered by non-specialist workers are designed to provide psychosocial support rather than comprehensive treatment for these problems. For some, returning to this MHPSS service reflected structural realities rather than a preference, as it was the only available treatment option or a necessary pathway for referral to specialist care. Across these accounts, participants intended to return not because their needs were met or they could more effectively deal with their problems, but because they felt respected, listened to and supported by practitioners they had come to trust.

In exploratory secondary analysis, the CSQ-4 had better psychometric properties, with all fit indices meeting established cut-offs. This reduced instrument removes item 4, which displayed gender-related measurement non-equivalence, capturing concerns about community stigma rather than dissatisfaction. Consistent with this, lower satisfaction scores observed in female participants when using the CSQ-8 were not observed with the CSQ-4. These findings suggest the CSQ-4 is a more coherent and robust measure in this context and could reduce gender- and stigma-related response bias. Nevertheless, the presence of item 8 in the CSQ-4 means large ceiling effects remain, potentially limiting the instrument’s ability to detect meaningful change. To our knowledge, despite its widespread use in global mental health research, the CSQ has not been culturally adapted or psychometrically evaluated outside of high-income settings. Further research is needed to adapt and validate satisfaction measures and other indicators of patient-reported quality of care globally.^11^

Convergent validity with the WAI-SR supports the central role of therapeutic relationships in shaping service satisfaction. This finding is consistent with previous qualitative work in this population, which highlighted the importance of relational and interpersonal aspects of MHPSS service delivery,^37^ and with the wider literature linking satisfaction to the quality of client-practitioner relationships.^9^ In this sample, the agreement on task and goal components of therapeutic alliance showed stronger associations and larger loadings than bond between service user and practitioner. Several factors may explain this pattern. First, items in the CSQ reflect expectancy theory (e.g. ‘Did you get the kind of service you wanted?’) and utility-orientated understandings of satisfaction (e.g. ‘Have the services you received helped you to deal more effectively with your problems?’). This may privilege practical help, collaborative tasks, and goal attainment over connection with the practitioner. Second, all services were delivered by non-specialist MHPSS workers. These models of care typically provide brief, structured psychosocial interventions, alongside practical, problem-focused support, which may mean agreement on goals and tasks are more prominent in MHPSS service users’ evaluations of care. Finally, the concept of therapeutic alliance may not capture the culturally specific ways relational trust, respect, safety and bonds are experienced, expressed or develop between Syrian practitioners and service users.

Global initiatives to improve quality of care in low- and middle-income countries have emphasised the importance of including service user voices in health system strengthening and quality improvement.^3^ Robust, contextually relevant measures of patient satisfaction should form part of these efforts. A recent study proposed a willingness to recommend a health facility as a more reliable indicator of user-reported quality than traditional measures of satisfaction.^3^ Similarly, recommending a service to others and planning to return to a service are frequently used to assess criterion validity in patient satisfaction measures.^38^ Our findings suggest this may be problematic in humanitarian settings or other low-resource contexts, where few services are accessible, available or affordable, and service users have little or no choice of provider. In our sample, items about recommending or returning to a service captured structural constraints on service users rather than satisfaction, and showed poor psychometric properties, limiting their discriminative power and utility as indicators of satisfaction. As the integration of MHPSS into primary care and community services is increasingly promoted to avoid medicalising social problems, reduce stigma, and promote continuity of care,^26,39^ the concept of returning to a single provider may not always be relevant.

### Limitations

Recruiting participants currently engaged with MHPSS services likely introduced selection bias and contributed to the high satisfaction scores. Our sampling strategy may over-represent individuals who continue attending services and under-represent those who disengage early, which might reflect dissatisfaction, unmet needs or structural barriers to attendance. Given the limited availability of services and substantial mental health needs in North-West Syria, participants’ evaluations of care may be influenced by social desirability and acquiescence biases. Concerns about damaging relationships with providers or compromising access to care, alongside a history of authoritarianism and low trust in health systems and institutions in Syria, may discourage participants from reporting dissatisfaction. While the delivery of services by local healthcare workers can improve access and coverage in humanitarian settings,^40^ it may also heighten concerns about privacy and confidentiality. Qualitative responses strengthened the interpretation of our findings. However, structured telephone interviews were the only feasible data collection method in a conflict-affected setting, and we were unable to conduct full cognitive interviewing. Telephone recruitment may have excluded people who do not have their own mobile phone, such as older people and women, and the decision to not contact potential participants if confidentiality could not be assured may have disproportionately affected women. Nevertheless, in conflict-affected regions like North-West Syria, remote data collection enables the participation of populations who would otherwise be excluded due to insecurity and other access barriers.^41^ The CSQ-4 was not administered as a standalone instrument. Instead, scores were calculated from a subset of CSQ-8 items, meaning these results should be regarded as exploratory. Finally, convergent validity could be examined only in a subset of participants who completed both the WAI-SR and CSQ-8. Although we observed moderate to strong correlations, the WAI-SR has not been validated in this population, so these findings should be interpreted cautiously.

In conclusion, this study highlights the value of combining psychometric and qualitative data to assess the validity and contextual interpretations of satisfaction measures. High satisfaction scores may overstate service quality, particularly in low-resource settings where expectations are low and options are limited. In this context, the CSQ-4 may be a more coherent and robust satisfaction measure than the CSQ-8. Items about a willingness to recommend and or return to a service should not be treated as straightforward indicators of satisfaction without considering local context.

## Supporting information

10.1192/bjo.2026.11013.sm001 McGrath et al. supplementary material McGrath et al. supplementary material

## Data Availability

Data are not publicly available to protect the privacy of research participants. Data may be available on request from the corresponding author, subject to relevant ethical approvals.

## Supplementary material

The supplementary material is available online at https://doi.org/10.1192/bjo.2026.11013
